# Cultural Evolutionary Tipping Points in the Storage and Transmission of Information

**DOI:** 10.3389/fpsyg.2012.00569

**Published:** 2012-12-19

**Authors:** R. Alexander Bentley, Michael J. O’Brien

**Affiliations:** ^1^Department of Archaeology and Anthropology, University of BristolBristol, UK; ^2^Department of Anthropology, University of MissouriColumbia, MO, USA

**Keywords:** cultural transmission, information, mode, tempo, tipping points

## Abstract

Human culture has evolved through a series of major tipping points in information storage and communication. The first was the appearance of language, which enabled communication between brains and allowed humans to specialize in what they do and to participate in complex mating games. The second was information storage outside the brain, most obviously expressed in the “Upper Paleolithic Revolution” – the sudden proliferation of cave art, personal adornment, and ritual in Europe some 35,000–45,000 years ago. More recently, this storage has taken the form of writing, mass media, and now the Internet, which is arguably overwhelming humans’ ability to discern relevant information. The third tipping point was the appearance of technology capable of accumulating and manipulating vast amounts of information outside humans, thus removing them as bottlenecks to a seemingly self-perpetuating process of knowledge explosion. Important components of any discussion of cultural evolutionary tipping points are tempo and mode, given that the rate of change, as well as the kind of change, in information storage and transmission has not been constant over the previous million years.

## Introduction

In Kurzweil’s (2005) best-selling book, *The Singularity is Near*, the well-known inventor and futurist predicts that by the year 2040 people will be able to upload their brains onto computers – just one of many incredible changes portrayed for popular audiences in Barry Ptolomy’s 2009 movie, *Transcendent Man*. Are Kurzweil and other popular futurists crackpots, or are they seeing something that many of us are not, even if some of the conclusions they draw are improbable at best? We would argue that if nothing else, they are drawing attention to a subject that is often overlooked in the social sciences, the future trajectory of cultural evolution. Those of us with a scientific interest in the evolution of culture (e.g., Mesoudi et al., 2004; Shennan, 2009; Whiten et al., 2011; Perreault, 2012) do well when discussing the past, but are we able to address the most poignant uncertainty of cultural evolution, namely, where might humanity be headed?

Here we pick up on that topic, using as a framework the concept of “tipping points,” a term made popular through Gladwell’s (2000) book of that title but which has well-established roots in sociology (e.g., Grodzins, 1958) and wide modern currency in climatology (e.g., Lenton et al., 2008; Russill and Nyssa, 2009; Lenton, 2011; Barnosky et al., 2012; Huntington et al., 2012). As Lamberson and Page (2012) point out, however, the term often is used colloquially to refer to unspecified “milestones” in such things as the economy (Hauser, 2011), markets (Ellison and Fudenberg, 2003), fashion (Gladwell, 2000), and the like, usually in concert with perceived sudden upturns in logistic (S-shaped) curves. As earnest as these applications are, they are wide of the mark in terms of what a tipping point is. We use the term more in the climatological sense, defining it as a critical threshold at which a tiny perturbation qualitatively *and irreversibly* alters the state or development of a system. This alteration occurs at some time downstream – perhaps well downstream – of the perturbation. The alteration may (or may not) manifest itself as a discontinuity on a logistic curve, depending on the scale at which the curve is plotted (Lamberson and Page, 2012).

We certainly are not the first to identify critical points in human evolution (e.g., Tobias, 1991; Johanson and Edgar, 1996; Modis, 2002), nor are such identifications restricted to the recent decades. The writings of Enlightenment scholars – Locke, Diderot, Rousseau, Voltaire, Montesquieu – are replete with examples of cultural “betterment” that eventually “tips” humans irreversibly to the next level of development. Montesquieu, for example, divided early mankind into savages and barbarians, and Turgot proposed a three-phase system of hunting, pastoralism, and agriculture. Later, the cultural evolutionists of the nineteenth century developed elaborate evolutionary schemes to pigeonhole ethnic groups and to identify the necessary cultural traits that “caused” a group to eventually move up from, say, savagery to barbarism (e.g., Morgan, 1877). Later still, archeologist Childe (1936) drew up a list of criteria that a group had to possess to be transformed into a “civilization,” among which were the plow, metal smelting, draft animals, writing, a calendar, irrigation agriculture, specialized craftsmen, and urban centers. A contemporary scheme by anthropologist White (1943) focused on the harnessing of more and more energy – first human muscle, followed by domesticated animals, plants, natural resources (coal and gas), and nuclear – as the catalyst for tipping points in cultural evolution. Not surprisingly, what grew out of these schemes was a cascade of supposed “revolutions” – the “Neolithic Revolution” (Childe, 1925), the “Urban Revolution” (Childe, 1950), and the “Agricultural Revolution” (Allen, 1999), among many others (e.g., “commercial,” “intellectual,” “political,” “industrial,” and “transportation” revolutions (see Ross and Tontz, 1948).

As important as these transitions undoubtedly were in the evolution of culture, we adopt a different view in that we concentrate on only two key features – information storage and communication. We see all other features as derivative. We identify three tipping points on the trajectory of human cultural evolution. The first was the appearance of language and the cognitive capabilities that accompanied it, which enabled hominins not only to “live inside their heads” (Brooks, 2010, p. 3) but also to communicate rapidly and reliably with other hominin brains. The second tipping point was information storage outside the brain, most obviously expressed in the “Upper Paleolithic Revolution” – the sudden proliferation of symbolic and technological complexity that occurred in western Eurasia some 45,000 years ago (Powell et al., 2009) and sporadically in sub-Saharan Africa even earlier (Jacobs et al., 2008). Later, information storage took the form of writing, mass media, and now the Internet, which is arguably overwhelming any ability to discern relevant information (Hemp, 2009). The third tipping point was the appearance of technology capable of accumulating and manipulating vast amounts of information outside humans (Donoghue, 2008), thus removing them as bottlenecks to a self-perpetuating process of knowledge explosion. In essence, the current stage of information technology is simply a waypoint in a long process that began well over a million years ago and that was driven by the interaction of genes and culture.

## Genes and Culture in Human Evolution

Social scientists have long known the power that culture exerts in shaping the human condition (Tylor, 1871; Wissler, 1923; Kroeber, 1952; White, 1959), but it is becoming increasingly clear that the interactions of genes and culture – literally, their coevolution – offer a faster and stronger mode of human evolution than either does by itself (Durham, 1991; Ehrlich, 2000; Richerson and Boyd, 2005; Laland, 2008; Laland et al., 2010; Richerson et al., 2010; Ihara, 2011; Rendell et al., 2011). Gene–culture theory is a branch of theoretical population genetics that incorporates cultural traits into models of differential transmission of genes from one generation to the next (Cavalli-Sforza and Feldman, 1981; Boyd and Richerson, 1985; Feldman and Laland, 1996; Richerson and Boyd, 2005; Laland et al., 2010; Richerson et al., 2010). The two inheritance systems cannot always be treated independently because (1) what an individual learns may depend on his or her genotype expressed throughout development and (2) selection acting on the genetic system may be generated or modified by the spread of a cultural trait (O’Brien and Laland, 2012). *Culture* is treated as information – for example, knowledge, beliefs, and skills – that is capable of affecting the behavior of individuals and which they acquire from other individuals through any of a number of social learning pathways, including teaching and imitation (Laland, 2004; Richerson and Boyd, 2005).

Gene–culture theorists model cultural transmission as a Darwinian process in which there is selective retention of favorable cultural variants (Boyd and Richerson, 1985), with accompanying effects on biological fitness. Recognition is given to the fact that other, non-selective processes such as mutation (invention, innovation), spread (diffusion), and drift (random change) play significant roles as well (Shennan, 2002; Bentley et al., 2004). Multiple animal species are able to learn, and a good number of them exhibit evidence of processes important to cultural transmission (Heyes, 1994, 2012; Galef and Laland, 2005; Laland and Reader, 2010; Whiten et al., 2011; Nielsen et al., 2012), but it is the fact that human culture evolves quickly and is cumulative (Enquist et al., 2011) that makes it an exceptional case. By this we mean that one generation does things in a certain way, and the next generation, instead of starting from scratch, does them in more or less the same way, perhaps with slight modification or improvement. The succeeding generation learns the modified version, which then persists across generations until further changes are made (Richerson and Boyd, 2005; Tennie et al., 2009). Cultural transmission is thus characterized by the so-called “ratchet effect,” in which modifications and improvements stay in the population until further changes ratchet things up again (Tomasello et al., 1993; Tomasello, 1999).

Selection pressures derived from culture can be stronger than non-cultural ones for at least two reasons. First, because there is highly reliable transmission of cultural information between individuals, culturally modified selective environments can produce unusually strong natural selection that is directionally consistent over time (Bersaglieri et al., 2004). Second, cultural innovations tend to spread more quickly than genetic mutations because social learning usually operates at a much faster rate (Feldman and Laland, 1996; Perreault, 2012). If cultural practices modify selection on human genes, the more individuals exhibiting a trait, the greater the intensity of selection will be on a gene (Laland et al., 2010). Gene–culture coevolutionary models repeatedly demonstrate more-rapid responses to selection than conventional population-genetic models, which helps explain the argument that culture has accelerated human evolution (Hawks et al., 2007). Conversely, under different circumstances, culture can also slow down genetic change (Feldman and Laland, 1996).

Gene–culture theorists face challenges similar to those of climate modelers when it comes to looking into the future. Both rely on the past for data points. Whereas climatologists tune their forecast models using ice cores and other ancient paleoclimatological records, gene–culture theorists rely on past millennia of gene–culture evolution as some guide to where the future leads (Laland et al., 2010; Richerson et al., 2010; O’Brien and Laland, 2012). This is reasonable, but there is a caveat: as good Bayesian reasoners, we may misunderstand the oncoming rush of the future because the past has sluggishly dragged on for the vast temporal portion of human evolution (Jones and Love, 2011)^1^. But if we look at the prehistoric past and compress its slow timescale, we see that the mind-boggling sci-fi possibilities of the present and future have prehistoric precedents. This compression, or logarithmic scaling, is common in the sciences. For example, John B. Sparks used it to create his spectacular one-page *Histomap of Evolution*, pointing out that “the most recent periods of evolution hold the most interest for us. We need therefore increasingly more space for our outline the nearer we approach modern times, and the logarithmic scale fulfills just this condition” (Sparks, 1932, p. 1).

Sparks was correct: by logarithmically scaling human evolution, we begin to see some of the prehistoric precedents (Cavalli-Sforza et al., 1994). The elimination of fear by modern neuropharmacology (Quirk and Mueller, 2008), for example, might seem a *Brave New World* (Huxley, 1932) possibility, but fear-reducing drugs have been common for decades, not to mention the fact that alcoholic drinks had a Neolithic origin (Dietler, 2006). The onset of the Neolithic around 11,000 B.C. (Bellwood, 2005) was, in fact, one of the more dramatic periods in gene–culture coevolution, as humans and their domesticated plants and animals began to coevolve on a millennial timescale (Zeder et al., 2006). In archeological sequences across the Northern Hemisphere, the emergence of agriculture coincides with a noticeable increase in artifactual remains, which has long been interpreted as indicating a spurt in demographic growth. Hemispheric cemetery data provide direct evidence of a major demographic shift characterized by a relatively abrupt increase in the proportion of 5- to 19-year-olds in cemeteries during the economic transition from foraging to farming (Bocquet-Appel, 2011) – a phase referred to as the “Neolithic Demographic Transition” (Bocquet-Appel, 2002). The world’s population just prior to the emergence of agriculture was perhaps around six million individuals (Biraben, 1979), compared to almost seven billion today, multiplying by 1200 in 11,000 years (Bocquet-Appel, 2011). The genetic changes that accompanied the rise of agriculture – and they were numerous, as we are continually discovering (Cordain et al., 2005; Perry et al., 2007; Gibbons, 2009; Pickrell et al., 2009; Laland et al., 2010) – were effected by the knowledge, specialization, and inequality in human societies, as well as by the densities of people who began living in villages and eventually in cities (Cochran and Harpending, 2009).

In modern times, the rate of gene–culture coevolution is poised to accelerate even more dramatically as humans begin to direct their own biological evolution through ever-increasing means of horizontal cultural transmission (Brosius, 2003; Hawks et al., 2007; Laland et al., 2010). One cultural source of this change is modern genetic engineering, which in this century may, for those who can afford it, lengthen life spans potentially by decades, eliminate genetic diseases screened before birth, and enhance human strength (Carlson et al., 2009) and possibly intelligence (Sisodiya et al., 2007; Bostrom and Sandberg, 2009; Cheng and Lu, 2012).

Many rapid changes that humans face now, and undoubtedly will in the future, are rooted in much slower changes that took place in the past. In discussing these, we distinguish between the *tempo*, or rate, of change and the *mode*, or kind, of change that took place. Important in all of this is recognizing that cultural evolution can be examined at myriad scales, and the scale at which we happen to be operating at any particular moment conditions our perception^2^. Analytically speaking, this is both a good and a bad thing. Various levels of view allow us to gain different perspectives on change, but taken singly, they lull us into thinking that one view represents the totality of change. For example, evolution is often presented as large-scale change that takes place over a long period of time. Such a presentation, although not incorrect, is only part of the story. Missing is the fact that the large-scale evolutionary results that we see so plainly are the cumulative products of countless smaller-scale, and hence much less evident, changes that occur continually. Yet by themselves, minute changes don’t constitute the entire story either because they leave out how changes in one part of a system affect the operation not only of the entire system but also of downstream systems far removed temporally – a result often referred to as “ecological inheritance” (Odling-Smee, 1988). Ecological inheritance does not depend on any kind of “replicator” but rather on intergenerational persistence (often through repeated acts of construction) of whatever physical – or, in the case of humans, cultural – changes are caused by ancestral organisms in the local selective environments of their descendants (Odling-Smee, 2010). Population-genetic models demonstrate that this ecological inheritance can generate unusual evolutionary dynamics (Laland et al., 2000, 2001; Ihara and Feldman, 2004; Borenstein et al., 2006; Silver and Di Paolo, 2006).

## Tipping Points: Changes in Tempo and Mode

Examining gene–culture interaction at various levels can lead to the detection of rapid changes in evolutionary tempo that might be signaling changes in mode as well (Laland et al., 2010; Richerson et al., 2010; O’Brien and Laland, 2012). The ethnological and archeological records are replete with evidence that the tempo of cultural change is rarely constant, although there are few cases in which it has been measured directly. How are scale and tempo correlated? Is the apparent rapid emergence of a new form actually sudden or is it an illusion, meaning that the scale at which we are examining something makes it appear as if the object is new when in actuality it is the product of myriad small-scale cumulative modifications that took place over a relatively long period of time? Stommel diagrams (fn. 2) suggest a positive relationship exists between tempo and scale in the sense that the faster the tempo, the smaller the scale (e.g., Westley et al., 2001).

This same kind of question was asked in paleontology for decades. Darwin’s notion of the evolution of species was based on gradualism – the slow build-up of small-scale change over geological time – although his theory did not require that tempo. Simpson (1944) opened the door on the notion of accelerated tempo, and Eldredge and Gould (1972) opened it wider with their concept of *punctuated equilibrium*. They argued that *cladogenesis* – the division of a taxon into itself and at least one sister taxon – is the general mode under which evolution operates (as opposed to *anagenesis*, or the evolution of one taxon into another) and that rapid cladogenesis is orders of magnitude more important than gradualism as a tempo of speciation.

In the hominin archeological record, irrespective of the scale at which it is being viewed, we would be hard pressed to see any tipping points in terms of information storage and retrieval in the one and a half million or so years that bifacially chipped Acheulean hand axes were used by Lower and Middle Paleolithic hominins across first Africa and later Europe (Scott and Gilbert, 2009; Lepre et al., 2011). Change, yes (e.g., Vaughan, 2001), tipping points, no. Maybe, to take a page out of Kurzweil (2005, pp 10–11), to those hominids their future was “pretty much like their present, which had been pretty much like their past.” But just as surely, all the slow changes in stone-tool technology that occurred over hundreds of thousands of years led to an accumulation that eventually exploded across Europe ca. 45,000 B.C., a point usually referred to as the beginning of the Upper Paleolithic.

That will be the second tipping point that we examine, and it is worth pointing out that it highly visible because of the nature of the phenomena that herald the change in tempo and mode. Cave art, ornaments, and tools made from antler and stone, which were part of the Upper Paleolithic Revolution (Bar-Yosef, 2002), are physical elements and therefore preserve rather well. This visibility stands in stark contrast to language, which does not fossilize (Hauser et al., 2002). We identify it as a tipping point in terms of information, but the timing of its occurrence is not well documented. As a result, it is more difficult to assess the tempo of the effects.

Returning to the accumulation of technological change, studies of modern material culture have found that inventive activities are discernible as a clustering in time and space of similar inventions – literally, a “burst of variation,” termed *stimulated variation* (Schiffer, 1996). Often, these bursts are associated with underlying technological or social changes that make possible new approaches to mitigating perceived deficiencies in products – a cultural process Schiffer (2005) labeled as the *cascade effect*. Changes in the context of cultural transmission, including the introduction of new cultural traits or shifts in previously unrelated or marginally related cultural traits, fundamentally alter tool traditions and their selective environments. This creates new adaptive spaces in which tool traditions change in response to new selective pressures (O’Brien and Bentley, 2011). A particular temporal dynamic goes hand in hand with stimulated variation. Initially, variation increases rapidly, as artisans experiment with designing effective forms – the bursts. Subsequently, variation decreases slowly, as less-efficient variants cease to be replicated (Lyman et al., 2008, 2009).

Rarely made explicit in models of culture change is the size of a population, yet clearly the more potential inventors and clever assemblers there are, the higher the number of solutions to problems there will be and the more chances there are for solutions to be copied and spread (Jones, 2001; Henrich, 2004; Powell et al., 2009; O’Brien and Bentley, 2011). Malthus (1798) understood that cultural change is intimately tied to the fact that population increases exponentially, whereas the food base increases only arithmetically. Ultimately, excessive demands on carrying capacity yield a dramatic decline in population correlated with cultural phenomena (including wars). Of course, the purely Malthusian approach leaves out the cumulative, and possibly exponential, effect of more minds working on communal solutions to problems that are more than simply the sum of individual parts. Through this distributed-mind approach – in essence, the creation of a super brain (Hoffecker, 2013) – we equate collective knowledge capacity with population size, rendered in terms of the number of people communicating and storing their specialized ideas.

The distributed-mind approach has had philosophical and empirical ramifications (Bolender, 2007; Hausmann and Hidalgo, 2011). Philosophically, it suggests that population size has a direct bearing on thought because what goes through an individual’s mind is largely derived from other minds. Thus an individual’s thoughts are but a sample of what is being thought around that person. Empirically, it means that the tempo of cultural change – partly a function of how much cultural information is preserved and passed on – may, in most cases, depend on population size (Powell et al., 2009). Technologically, it means that a modern economy serves the role of disseminating, aggregating, and sorting the technological capabilities, yielding a highly non-linear correlation between the number of technological capabilities in a country and the number of products it can make (Hausmann and Hidalgo, 2011) and/or between economic complexity and per capita income. As we discuss later, an exciting new body of work posits that increasing population size rather than changes in biological ability may underlie some of the major shifts seen in cultural prehistory, such as the Upper Paleolithic Revolution (Powell et al., 2009) or the much-debated cultural capacities of late Neanderthals (Zilhão et al., 2010). Conversely, population bottlenecks are now proposed to reduce cultural knowledge collectively, as Henrich (2004) has argued convincingly for prehistoric Tasmania.

Just as more people are alive now than have ever lived on Earth before, more technological change has occurred in the last decade than cumulatively over the history of humanity (Beinhocker, 2006). We can certainly agree with Kurzweil (2001, 2005) that the tempo of cumulative technological change has grown exponentially over the last century. Following the well-known Moore’s Law (Moore, 1965) for the doubling of computing power (expressed as calculations per second per dollar, or alternatively as the number of transistors that fit on a silicon chip) every 2 years over four decades, Kurzweil (2001) demonstrated exponential growth in brain-scanning speed, the cost of DNA sequencing, the number of human genes mapped per year, and the shrinking size of technology. His predictions have proven remarkably resilient.

Like Malthus, Stephen Pinker knows that exponential trends cannot be extrapolated indefinitely, declaring that genetic enhancement is not inevitable, nor is it particularly likely in our lifetimes (Pinker, 2003). All exponential change has to level off eventually, of course (Mesoudi, 2011b), often in the form of a logistic curve exhibited by population growth or the diffusion of new technologies (Rogers, 1964). The question, however, is *when* change levels off, as the exponential nature of technological change has arguably been true for thousands of years in cultural evolution (Beinhocker, 2006). In technological evolution, these logistic curves can occur more or less sequentially, or with some overlap, as the pace of change is punctuated.

How do we know when a curve will decelerate? Ideally, we would extrapolate from observed exponential growth up to the present into the full logistic curve of the future. The classic cultural-diffusion model represents the probability of the adoption of a technology at time *t* as

p(t)=(μ+qF(t))(1-F(t)),

where the parameters μ and *q* represent the degree of innovation and imitation, respectively, with the assumption that μ << *q*. The cumulative function yields the logistic curve, such that the rate of adoption increases exponentially at first, then reaches a peak, and then declines as the cumulative number of adopters asymptotically approaches a saturation of the population of potential adopters (Brock and Durlauf, 2001, 2010; Lamberson and Page, 2012).

This model, however, applies to the *diffusion* of innovations, not to the *creation* of innovations, and hence tells us little about the future of the exponential innovation rate in technology (Bentley and O’Brien, 2011). Another problem, as Lamberson and Page (2012) point out, is that exponential change is a smooth, continuous acceleration, not a form of punctuated change or tipping point. More-accurate signs of tipping points are reviewed by Scheffer et al., 2009; see also Drake and Griffen, 2010; Scheffer, 2010) as “early warning signals” for critical transitions, or true tipping points that lead to new forms of behavior. With respect to the time series of events, these signals include slower recovery from perturbations, unequal rates of recovery in the different directions toward equilibrium, and increased autocorrelation, variance, and “flickering” in the time series.

Aside from this list of warning signals, we can consider some tipping points of the past, including the very recent past, where the mode of gene–culture evolution changed dramatically through new forms of information transmission. We divide these into three major modes. In the first, hominins became able to store and manipulate information, arguably about kinship relations, within themselves. They did it in a manner that determined the treelike organization of information storage in human minds. In the second, humans began to store information outside themselves in art, material culture, and later writing and now the Internet. This allowed for an accumulation of information, but more important, individuals began linking their stores of knowledge to those of others, made possible by changes in social organization. In the third phase, new knowledge is created outside human minds by searching, manipulating, and combining accumulated information through artificial intelligence.

## Tipping Point 1: Communication Between Brains

Among hominins, gene–culture coevolution has accelerated for millennia, not necessarily steadily but in successive minor tipping points that ushered in new magnitudes in the rate of change. Looking back roughly two million years, the pace of this coevolution would be gauged on the scale of hundreds of thousands of years, as our digestive systems gave way to larger brains (Aiello and Wheeler, 1995; Wrangham, 2009). Consequently, our social organization allowed for a division of labor and a shared caring of newborns, which is unique among primates (Hrdy, 2009). At some debatable date – estimates vary from over a million years ago to just 45,000 years ago – our brains enlarged, which the evidence suggests facilitated engaging in social relations (Alexander, 1989, 1990; Dunbar, 1996; Bailey and Geary, 2009). Flinn et al., 2005, p. 14) view the evolution of this “social brain” (Dunbar, 1998, 2003) as the result of “the diminished intensity of selection from extrinsic causes compared with the relative importance of selection from interactions with conspecifics.” Integral to this social engagement were signs, gestures, and eventually language.

Speech production was not a sudden phenomenon but rather a point in a long process of co-opting preadaptations in the jaw, tongue, larynx, and hypoglossal canal (Denes and Pinson, 1993). Evidence suggests that the evolution of breathing control and increased brain size could have enabled complex communication by the time of the last ancestor common to both Neanderthals and modern humans at about 600,000 years ago (Krings et al., 1997; MacLarnon and Hewitt, 2004). Certainly both Neanderthal and modern human populations shared the derived form of the FOXP2 gene (Krause et al., 2007), which has been implicated in the development of speech and language (Enard et al., 2002). But having the capacity for complex communication and actually using it are two very different things. Language, as we noted earlier, leaves no fossil clues, which is why dating the origin of language has been referred to as “the hardest problem in science” (Christiansen and Kirby, 2003). That said, statistical analyses of linguistic diversity and divergence are beginning to suggest an age of roughly 100,000–200,000 years for the origin of language (Nichols, 1998; Perreault and Mathew, 2012), which makes it contemporaneous with the appearance of *Homo sapiens* as a species (Botha and Knight, 2009). Similarly, detailed analysis of the distributions of personal items such as beads from archeological sites across Europe suggests that ethno-linguistic boundaries can be approximated for some Upper Paleolithic cultures (Vanhaeren and d’Errico, 2006).

The advantages of language, as the beginning of information storage in the minds of others, allowed hominins to live, hunt, gather, and groom in groups and to gossip in the mating competition (Dunbar, 1996). By providing “much more efficient channels of communication… language apparently increased the potency of human cultural processes by orders of magnitude” (Odling-Smee, 2006, p. 53). Although Chomsky (2002) and others (e.g., Pinker and Bloom, 1990) have argued that the existence of ambiguity in language proves that the structures and properties of language did not evolve for purposes of communication, it is now clear that ambiguity is a desirable property, precisely because it allows for a communication system that is “short and simple” (Piantadosi et al., 2012, p. 281).

In studies of the evolutionary origins of human language (e.g., Hauser et al., 2002; Corballis, 2011), *recursion* has often been proposed as the central organizing principle that separates human language from all other animal communication systems. Recursion is a multi-definitional phenomenon (Fitch, 2010; Martins, 2012), but for our purposes here we can define it as “the ability to take a finite set of ideas and link or embed them to form an infinite array of thoughts, phrases, or expressions” (Gallagher, 2011, p. 229), or, simply, the ability to place thoughts within thoughts within thoughts (Corballis, 2011), similar to nested Russian dolls. Recursion is both ubiquitous in human languages and unique to humans. Indeed, recursive syntax of human language may underlie its semantics (Hinzen, 2007); this hierarchical compositionality is what enables human cognition. If recursion proceeds from the hierarchical structure of neuronal activity (e.g., Becker and Flaxer, 2008), it follows that some treelike organization of information retrieval in the human brain preceded language evolution (Hoffecker, 2013). In fact, there is evidence that other animals, including honeybees (Gould and Gould, 1995) and baboons (Bergman et al., 2003), possess complex hierarchical mental structures.

The human brain is energetically expensive in comparison to those of non-human primates (Aiello and Wheeler, 1995; Navarette et al., 2011), and it takes a lot of energy to “think.” Thus, there have to be direct benefits from possessing a larger, more expensive brain, one of which appears to have been the ability to meet the cognitive demands of maintaining meaningful social interactions within increasingly larger groups (Byrne and Whiten, 1988; Dunbar, 1996, 1998; Dunbar and Shultz, 2007a,b). For a social brain, a central challenge, providing a corresponding benefit, is to keep track of eligible and non-eligible mates or protectors (Dunbar, 2009). This challenge is much more complex under any known human kinship system than in any other primate species. According to Hamilton’s (1964) kinship-selection theory, relatedness is a central parameter in the maintenance of social relations, cooperation, and mate choice (Cronk and Gerkey, 2007). In other words, such choices correlate with genetic proximity.

In a wide array of situations where proximity is a key organizing principle, one of the most efficient means of organization and distribution is through the use of treelike structures. Such structures can self-organize in biological systems and be refined by natural selection, as in the case of human vascular networks (West et al., 2002), neural networks (Becker and Flaxer, 2008), and the hierarchical group organization of social mammals (Hill et al., 2008). This hierarchical organization also characterizes hunter–gatherer groups, whose size versus frequency shows self-similarity over a range of scales (Maschner and Bentley, 2003; Hamilton et al., 2007). At the scale of individuals, the division of labor between men (hunting) and women (gathering) is reinforced pair bonding (Chapais, 2008; Hill et al., 2011; Walker et al., 2011). At a larger-scale, the band serves as a social unit that often resolves tension between dispersion and interdependence. At the wider community level, humans sustain social relations through gift exchange, marriage, and classificatory kinship.

Social primates such as baboons organize themselves through hierarchical classification derived from individual rank and kinship (Bergman et al., 2003; Hill et al., 2008), but because humans are unlikely to know all members of their community, organization is sustained by overlapping networks of kinship, marriage, and friendship. Longer-term planning and greater need for information about others’ intentions render language use highly adaptive in human social organization. Given that the numbers of kin to keep track of increase geometrically with genetic distance, and that kinship organization is central to human reproduction, it is not surprising that many human cultures organize knowledge of kinship descent into simplified systems of classification (Cronk and Gerkey, 2007; Jones, 2010; Kemp and Regier, 2012; Levinson, 2012).

As kinship systems serve a number of functions, they must be adaptable, with many possible states (Lyon, 2005). This “entropy” creates a challenge for explaining continuity (Sperber, 1985). Classification trees provide a flexible solution. Some, but not all, cultures have evolved to classify kin in treelike decent systems – “family trees” – but regardless of whether kinship concepts emphasize descent, affinity, generation, siblingship, or other features, graphical representations of the relations between kinship terms typically display a treelike form (Kemp and Regier, 2012).

As with the narrow language faculty proposed to be unique to humans (Hauser et al., 2002), kinship terminologies are intrinsically recursive, without reference to external social or biological phenomena (Read, 2001; Leaf, 2005). Indeed, the recursive generative principles of kinship terminologies allow modeling to predict the terminological positions of real kinship systems (Jones, 2010). Language allows us to store information in other people’s minds. As a result, the larger hominin groups became, the more information, including kinship information, could be stored in the collected minds of its individuals (Renfrew and Scarre, 1998; Powell et al., 2009). Alternatively, the smaller groups became, the more likely knowledge would be lost. We return to this point below.

Similar considerations of population size bring new insight, for example, into a fascinating current debate surrounding the Pirahã of Brazil, whose language arguably lacks recursion (Everett, 2005) and whose counting system appears rudimentary (Gordon, 2004). These conclusions are heavily debated (e.g., Levinson, 2005), but in the debate few have considered the fact that the Pirahã have lived in very small numbers (hundreds) since they broke from the larger native population of the Mura by the early eighteenth century.

## Tipping Point 2: Storage Outside the Brain

About 45,000 years ago much of western Europe witnessed a proliferation of cave art, personal adornment, and rituals – what archeologists refer to as the “Upper Paleolithic Revolution”(Bar-Yosef, 2002; Mellars, 2005). The roots of this proliferation appear to lie in migrations of anatomically and cognitively modern humans out of sub-Saharan Africa (Ambrose, 1998; Lahr and Foley, 1998; Ray et al., 2005), where there is intermittent evidence of symbols and personal adornment (e.g., shell beads and engraved chunks of ochre and ostrich egg shells) that in some cases dates several tens of thousands of years earlier (McBrearty and Brooks, 2000; Henshilwood et al., 2002, 2011; Mackay and Welz, 2008). Regardless, there is no denying the explosion in creative expression that occurred in western Europe around 45,000 b.c., coupled with the appearance of such features as long-distance exchange, grinding implements (Wright, 1992), and storage facilities, especially in northern latitudes where underground freezing kept food edible (Soffer, 1989). What caused the explosion? The answer may not be *what* was in humans’ heads but in *how* the heads, and how *many* heads, were interconnected.

Explanations for the Upper Paleolithic transition have long featured cognitive changes in the human mind (e.g., Mithen, 1996; Klein, 2002), but we disagree, as do others (d’Errico and Stringer, 2011; Foley and Lahr, 2011). Rather, we see signs that demographic changes in human populations – changes coincident with the arrival of groups out of Africa and their infilling of parts of Europe – were directly responsible for the Upper Paleolithic Revolution. Powell et al. (2009) used Henrich’s (2004) model of cultural loss in Tasmania, to which they added stochastic and geographic elements, to show how chance clusters of local migrating populations – similar to those in the Upper Paleolithic – could, by exceeding the crucial population threshold, begin to undergo cumulative cultural evolution over generations. The implications of these findings are radical. A larger *local* population meant that more people were around to invent new ideas, build on earlier ideas (the cumulative aspect of culture), and, crucially, pass on those ideas before they were lost. This population aggregation provided the critical “ratchet” (Tomasello, 1999) to push culture to a tipping point.

An increase in local population density would have been particularly relevant if *Homo sapiens* 45,000 years ago possessed mirror neurons that in part facilitated social interaction. The existence of mirror neurons in humans is now incontrovertible (Mukamel et al., 2010), and it is clear that they are not restricted to the premotor and inferior parietal cortex of the brain (Keysers and Gazzola, 2010). The question is when they appeared in the hominin line. Hodgson (2012, p. 358) concludes that the “mirror/theory-of-mind system… was already in place before the appearance of *H. sapiens* (ca. 200,000 years ago) and that the ebb and flow of the archeological record during the Middle to Upper Paleolithic reflects the various ecological conditions that affected behavior as function of this neurocognitive substrate.” Regardless of the timing, the relevance of local population size is inescapable: more people would have meant more potential role models to imitate in carrying out complex behaviors that were expensive to learn. Similarly, however, a decrease in population can lead to the loss of skills, particularly technological knowledge related to complex “tools that are hard to learn to make, and easy to screw up” (Henrich, 2006, p. 776).

We argue elsewhere (Bentley and O’Brien, 2011) that what Henrich (2004, 2006) and Powell et al. (2009) refer to as “cumulative adaptive evolution” is essentially a niche-construction model (Laland et al., 2000, 2001) with an assumed probability distribution of skill levels, which feed back on themselves through intergenerational learning, becoming more specialized as the accumulated skill level increases. Boyd et al. (2011) refer to this as the “cultural niche” – an interconnectedness of brains made possible only through social learning. The upside of social learning is enormous. In contrast to organisms that can learn only individually, even when it is expensive and error prone, social learners can tap the brains of others (Mesoudi, 2008, 2011a,b). At that point, they no longer have to know everything, they just have to know in whose brain the requisite information resides – Gamble et al. (2010) “social brain, distributed mind.” Thus social learners can “afford to be choosy, learning individually when it is cheap and accurate, and relying on cultural learning when environmental information is costly or inaccurate” (Boyd et al., 2011, p. 10921; see also Mesoudi, 2008). This raises the average fitness of the group as well because acquired improvements can accumulate from one generation to the next (Tomasello et al., 1993; Tomasello, 1999; Boyd et al., 2011; Perreault, 2012).

A key event that occurred in the Upper Paleolithic – beginning perhaps as early as 40,000 years ago (Pike et al., 2012) – was the storing of information on cave walls through painting (Leroi-Gourhan, 1982; Whitley, 2009). Common themes included human hands and large animals – aurochs, horses, and deer – that presumably were hunted. Cave painting in Europe continued for several tens of millennia, until at least the beginning of the Neolithic, around 11,000 B.C. Writing, which began in Mesopotamia roughly during the fourth millenium B.C. (Fischer, 2004), was used mainly as a form of book keeping. Later, more-expressive writing then became a specialist endeavor, meaning it was limited to a few, from the priests of Mesopotamia to the scribes entrusted with the Code of Hammurabi (Van de Mieroop, 2004). As writing became a means of creative expression – from the Greek tragedies, to the tales of Chaucer and the plays and sonnets of Shakespeare, and finally to the novels of the eighteenth and nineteenth centuries – writing became a means for the few to communicate with many, either through performance or later through readings by literary societies (Bradway-Hesse, 1998). In doing so, this “library” gradually became the hub of culture itself rather than just a means of expressing it. The intricately connected web of broadcast media that we see today is just the extreme of this trend.

Because of writing, the vast amount of specialized knowledge in the world is perhaps a billion times the technological variation contained in a prehistoric hunter–gatherer community (Beinhocker, 2006). This specialization has intensified incredibly, such that only a tiny fraction of people in the developed world knows how to produce food, and modern urbanites can often specialize in quite arcane knowledge that would have virtually no value for survival on one’s own or in the small groups of our ancient ancestors. Culture has, to this point, provided the means for even semi-isolated individuals to maintain a subsistence while simultaneously engaging in other pursuits.

Today, many of those pursuits revolve around the Internet, which in a way represents a return to the past, before mass media, by making local craft traditions and self-expression possible again through uploading personal videos, blogs, pictures, and social-network homepages. Cultural change has accelerated not because of the larger network but because Web 2.0 motivates many more people to create new ideas. The interconnectedness of online endeavors – through blogs and social-network sites as well as search tools such as Google and Bing – has not homogenized culture, as some feared. Rather, it has fractionated it, as like minds find each other to create cultural “niches” that branch off from one another. Interconnectedness, paradoxically, allows groups to differentiate by copying each other, which homogenizes the group but further distinguishes it from all other groups. Instead of family and political ties organizing people in geographic space, it is now ideas and common interests that organize people online.

This change in tempo necessarily brings about a change in mode of evolution because modern technology is no longer a set of knowledge that people can teach to the younger generation in the way craftspeople apprenticed their children or teachers taught pupils for millennia. The age-old vertical (intergenerational) transmission is not nearly fast enough anymore; instead, the majority of competitive technological knowledge *must* be transmitted horizontally. Horizontal transmission will change genetic evolution as well. Humans have entered a phase of evolution where not all phenotypic traits are inherited genetically from parent to offspring – witness the case with plastic surgery and the imminent and inevitable merging of humans with computer technology (neural-interface systems; Donoghue, 2008). It is also the case for traits that up until now were genetically inherited, such as strength, height, and intelligence, all of which look to become increasingly horizontally transmitted with genetic engineering (Bostrom, 2005).

## Tipping Point 3: Manipulating Accumulated Information Outside Humans

If information is easily located and strongly selected for utility, this should lead to fitness. In this sense, information technology thus becomes an “equalizer” (Ding et al., 2010) because it allows a larger proportion of the population to be involved in finding, linking, and generating knowledge – a necessary capability for modern national prosperity (Hausmann and Hidalgo, 2011). If, on the other hand, information overload makes it more difficult to select the correct information, no matter how easy it is to find, the result could be more sorting and more clique-based fractionation (Evans and Foster, 2011).

Information overload is not a recent phenomenon. Invention of the printing press and the rapid increase in the number of books suddenly available caused sixteenth- and seventeenth-century scholars to wring their hands over the situation (Rosenberg, 2003; Blair, 2010). What we see today, however, is exponentially more problematic. So much information is available online that even with ever-sophisticated search engines we no longer can peruse even a fraction of it (see Mesoudi, 2011b). The increase in quantity has meant that academics are prone to re-invent the wheel in isolated niches, and journal articles are hastily published, with careless review and even frequent typos (Simkin and Roychowdhury, 2003; Evans and Foster, 2011).

Modern social learning yields ubiquitous right-skewed “winner-take-all” distributions of wealth and popularity on a scale the world has never seen (Frank and Cook, 1996). Other day-to-day behaviors, however, still exhibit a regression to the mean (Kahneman, 2011), now demonstrated by big-data studies. Hence, even though the number of Twitter followers is heavily right-skewed (Cha et al., 2012), the actual textual contents of Twitter feeds converge on average experiences that are obvious by definition. New studies of Twitter and Facebook exemplify the unprecedented technological achievement of analyzing literally millions of human interactions, but so far they are yielding somewhat banal conclusions, such as that people awaken later on weekends (Golder and Macy, 2011) or that influential people with influential friends are good spreaders of information (Aral and Walker, 2012).

This is not a critique so much as an example of how “big data” computer scientists effectively have to re-invent aspects of social science from scratch when confronted with a literature so vast that no human being could possibly read and comprehend it, made all the more difficult because of different academic backgrounds. The overabundance of information is alleviated only as computers are programed not just to search through vast quantities of information but also to perform hypothesis-testing science with the information (King et al., 2009a). Humans, then, may not be leading the way in science, unless, of course, gene–culture coevolution leads them to merge with computers. In the extended-phenotype sense (Dawkins, 1990), this has already happened, as most in the Western world would not do as well today without computers facilitating their communication, wealth storage, and work. Even more striking, although perhaps not yet as significant in gene–culture terms, is the physical merging of human minds with computers, as exemplified on the cover of *Nature* with the “braingate” technology (Hochberg et al., 2006; Donoghue, 2008). Progress on the brain–machine interface continues from there (Hochberg et al., 2013) as researchers embark on the “long-term neurophysiological investigation in human cortex at the level of single and multiple neurons… which has previously never been possible” (Hatsopoulos and Donoghue, 2009, p. 252).

This leads to fascinating debates over whether science will soon become automated by computers (Bostrom, 2003; King et al., 2009a; Schmidt and Lipson, 2009), ultimately elbowing humans out of the very process they created. On the one hand, it may be that machines will never be capable of creative *exploration* of science and only of what Axelrod and Cohen (1999) called *exploitation*, or

what most scientists do most of the time – which [Thomas Kuhn] called ‘normal science’ and Rutherford called ‘stamp collecting’. [This] does not contribute very much to the advancement of knowledge; rather, this normal science simply fleshes out the consequences of the paradigms that have been established by truly revolutionary advances. Even if machines did contribute to normal science, we see no mechanism by which they could create a Kuhnian revolution and thereby establish new physical law (Anderson and Abrahams, 2009).

On the other hand, machine automation has already been used in chess, for example, where “there is a continuum in player skill, and computers slowly improved with advances in computer hardware and software until they now play at world championship level… [T]here is a similar continuum in the ability to do science, from what robot scientists can do today, through what most human scientists can achieve, up to the level of a Darwin or Newton” (King et al., 2009b, p. 945).

Humans have now created so much knowledge and skill outside their own bodies, in computers and information networks, that that knowledge may soon feed back on its own into this process. Anderson and Abrahams (2009) argue that progress in science requires true outliers – those creative geniuses that fit Thomas Edison’s inspirational one percent. Part of the debate is the mystery over the origin and timing of genius. Gladwell (2008) suggested that some geniuses, such as Cézanne, needed thousands of hours of practice before achieving the height of their skill, whereas others, such as Picasso and Einstein, made (arguably) their most profound achievements at a young age, with very little practice. Similarly, Galenson (2005) divided artists into two groups based on an assessment of when they made their greatest contributions – *conceptualists*, who are innovative at an early age, and *experimentalists*, whose innovations come much later, after considerable experimentation and refinement. Regardless, every so often an innovation comes along that is rare enough to begin a new paradigm in the true Kuhnian sense of the term (Kuhn, 1962). As soon as a good new idea is demonstrated, many clamber to copy it and modify it slightly. This alteration may underlie a continual budding-off process as new, more specialized niches are created and then developed (O’Brien and Shennan, 2010).

It might be better to view technology as an “autocatalytic” process (Farmer et al., 1986; Kauffman, 1995). Autocatalytic processes also generate logistic curves in terms of rate of change: slow growth at first, when there is little product to catalyze its own formation, then a rapid acceleration as the product increases and catalyzes its own formation, then a slowdown as all is converted. In terms of the acceleration stages, genetic research could be seen as possessing this “autocatalytic” feedback, for example, when a new method of mutating the entire genome (Wu et al., 2007) promises to boost the tempo of genetic research even further (because the effects of more mutations can be tested in a shorter time). Arthur (2009) recently elaborated on this idea in a book on how new technologies are assembled from pieces of existing technologies, such that technologies share common ancestries in the true evolutionary sense.

These are established concepts for evolutionary anthropologists interested in hierarchical analysis of the material record (e.g., Collard et al., 2008; Mesoudi and O’Brien, 2008; O’Brien et al., 2008; Shennan, 2011). The treelike form of phylogenetic representation makes it apparent how technological evolution is a similar branching process (Arthur, 2009) in which older, established technologies give rise to a generation of “spin off” technologies nested within them, which themselves give rise to another nested generation of spin offs, and so on. The evolution of ideas also reveals this nested pattern (Bentley and Maschner, 2000), but identifying the pattern does not explain when the next punctuated transition will occur.

## Discussion

Cultural evolutionists, especially those who ponder possible gene–culture trajectories of the future, are inexorably becoming more interested in how non-linear change is inherent to evolution. As in physics decades ago (Anderson, 1972), there is increasing recognition that the emergence of novel, large-scale cultural phenomena cannot be reduced to a series of subcomponents. What might loosely be referred to as *emergence theory* has made its way into biology (e.g., Reid, 2007) and philosophy (e.g., Wimsatt, 1997), although there clearly is a lack of consensus over what emergence entails and even open hostility toward it – witness John Maynard Smith’s remark that the study of complex adaptive systems is “fact-free science” (Horgan, 1995). Change is still slow, however, as the discussion in the social sciences approaches its own tipping point. In anthropology, emergence, with a few notable exceptions (e.g., Reynolds and Saleem, 2005), has been anchored in an orthogenetic reliance on progress as an explanation of cultural evolution: what humans need, humans get in order to make their way up the ladder of cultural complexity (O’Brien and Shennan, 2010).

Here, however, emergence has nothing to do with progress, nor is it simply a theory of hierarchical arrangements, whereby innovations can be scaled into components, subcomponents, sub-subcomponents, and the like. Rather, it signifies irreducibility in terms of scale. Much more so than is typical in biology, tempo and mode interact in cultural situations to create a new source of innovation at scales both large and complex – what Trigger (1998, p. 364) referred to as the “unique, emergent aspects of human behavior.” As Mesoudi and O’Brien (2008) point out, a “cultural evo-devo” would address the emergent properties of human behavior, focusing attention on the processes by which novelties (tools and other human behavioral products) are generated by culturally transmitted information stored in the brain and how this process interacts with macroevolutionary change (Eerkens and Lipo, 2005).

Following the development of punctuated equilibrium in the 1970s (Eldredge and Gould, 1972), tipping points were explored by social scientists and mathematicians interested in catastrophe theory (e.g., Thom, 1975). For example, spurred by an interest in the conversion to agriculture by hunting-gathering groups in the presence of colonizing farmers, Renfrew (1978) showed how, as external parameters (e.g., climate) change even very slowly, quite abrupt transitions can nevertheless take place in human cultural systems. Renfrew hypothesized a cultural system with several possible equilibria in which multiple variables affect benefit in contradictory, non-linear ways. Although individual behavior is changing only incrementally over the generations, this can reach a “bifurcation” point (Brock and Hommes, 1997; Brock and Durlauf, 2001), where it is suddenly necessary to make a drastic change in order to maintain the optimal behavior. Similarly, modern technologies characterized by “network externalities” or “external increasing returns” (e.g., Ormerod, 1998; Arthur, 2009) – such as mobile phones or perhaps someday even genetic engineering or cybernetics – often enter as mere novelties, but as the number of adopters gradually increases, a point of cost effectiveness can be reached such that the technology is then abruptly adopted en masse. Indeed, more than a decade ago, Cabral (1990) showed that network externalities could easily cause jumps in adoption rates. Similarly, Brock and Durlauf (2010) have a fairly extensive discussion of the effects of social interaction, which is a form of social-network externality, on the shapes of adoption curves.

## Conclusion

What can we take away from our brief foray into cultural evolutionary tipping points in the storage and transmission of information? We would offer the following. First, the pace of cultural evolution has accelerated over time, much of it tied to the ways in which humans have been able to harness and manage information. Second, although it is easy to be persuaded that there was some inevitability or direction to this harnessing and management, it is true only in a retrodictive sense. In other words, we see nothing in the archeological record that would have allowed us at any point in the past to predict what the future might entail in terms of information management. Third, of the three tipping points we discuss, perhaps the most significant was the ability to store, and tap, information outside one’s own brain, whether that information resides in the heads of others or on a cave wall. Fourth, the emergence of this ability was tied directly to local population size, meaning that more people increased the odds both of new ideas ratcheting up old ones and of passing those ideas on before they were lost. Fifth, tempo and mode are both important components of the myriad ways in which humans have stored and manipulated information, but it is clear that changes in tempo can bring about changes in mode of evolution.

Tempo and mode are only two of the myriad issues that have as yet been inadequately addressed with respect to the origin and spread of cultural innovation, yet they offer exciting entry points into the discussion (O’Brien and Lyman, 2000). Certainly they were central to much of the work undertaken in the field of paleobiology, which emerged in the 1940s, but they have been less so in anthropology. Whether one views punctuated equilibrium as a particularly useful model in understanding the origin and spread of innovation, there should be no denying that it calls attention to the inextricable linkage between tempo and mode and the creation of tipping points. It also highlights the issue of scale and the visibility threshold of innovations. And it appears to be a useful template for understanding not only the last 45,000 years or so of human gene–culture coevolution but future trajectories as well.

## Conflict of Interest Statement

The authors declare that the research was conducted in the absence of any commercial or financial relationships that could be construed as a potential conflict of interest.

## Footnotes

^1^Indeed, the dead hand of the past places so many past observations into the Bayesian updating mechanism over a long distant past that it essentially acts as if it almost ignores the near past, which sensible forecasters would use to forecast the future, especially when the environment is rapidly changing. This is sometimes called “panacea-like” behavior in other contexts (Brock and Carpenter, 2007), but it is relevant in any adaptive system with Bayesian-like updaters. Compressing the slow time scale of a long past can be viewed as a mechanism to put more weight on observations from the near past, which are more relevant for helping us peer into the future, especially in a rapidly changing world like the one at present.

^2^A useful schematic, the Stommel diagram, can be used to depict the common phenomenon of slower rates of change being positively associated with larger kinds of change (Westley et al., 2001). Stommel diagrams suggest a positive relationship between tempo and scale in the following sense: the faster the tempo the smaller the scale. Another way of putting it is that slower phenomena in time are associated with larger-scale phenomena in space, where “space” is widely interpreted.

## Author Note

R. Alexander Bentley is professor and chair of the Department of Archaeology and Anthropology, Bristol University, Bristol, UK. He recently published (2011) *I’ll Have What She’s Having* with M. Earls and M. J. O’Brien (Cambridge, MA: MIT Press). Michael J. O’Brien is a professor in the Department of Anthropology and dean of the College of Arts and Science, University of Missouri, Columbia, USA. He recently published (2010) *Innovation in Cultural Systems: Contributions from Evolutionary Anthropology* with S. J. Shennan (Cambridge, MA: MIT Press).
